# Supplementary material for: Allogeneic TCRαβ deficient CAR T-cells targeting CD123 in acute myeloid leukemia
Source: Nat Commun. 2022 Apr 28;13:2227. doi: 10.1038/s41467-022-29668-9 (PMC9050731; doi:10.1038/s41467-022-29668-9)
Supplement: Supplementary file 1 — Supplementary information [file 41467_2022_29668_MOESM1_ESM.pdf]

Supplementary Figure 1

a.

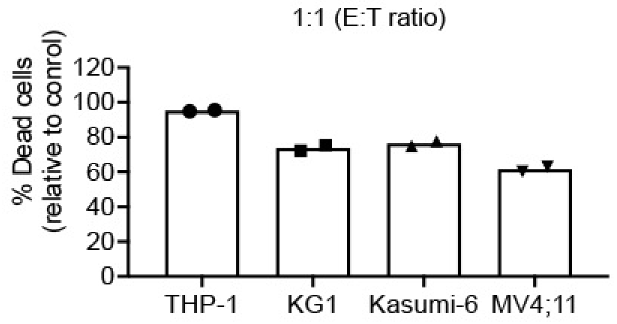

b. **CD5+ lymphocyte**

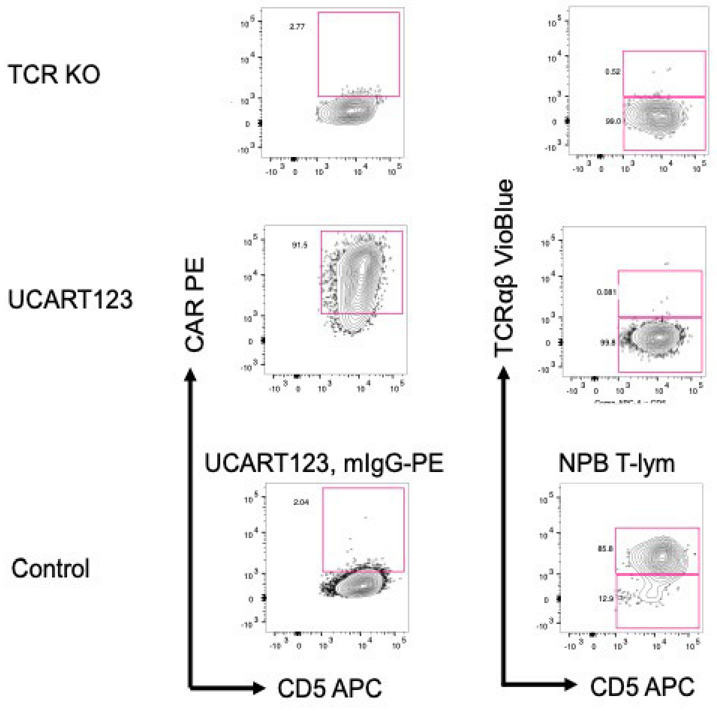

c. **Flow cytometry for Rituximab treatment**

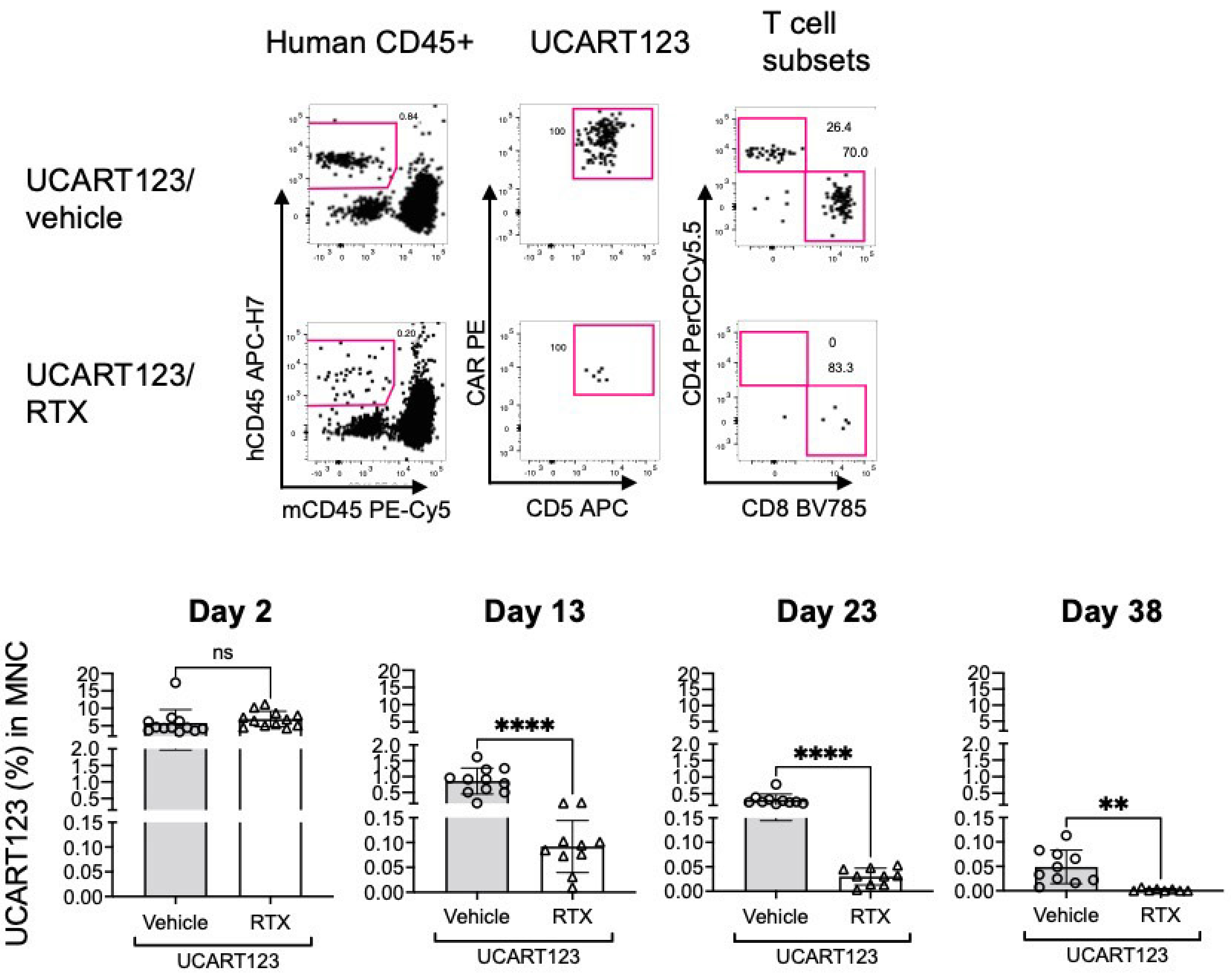

d. IVIS (leukemia burden) for Rituximab treatment

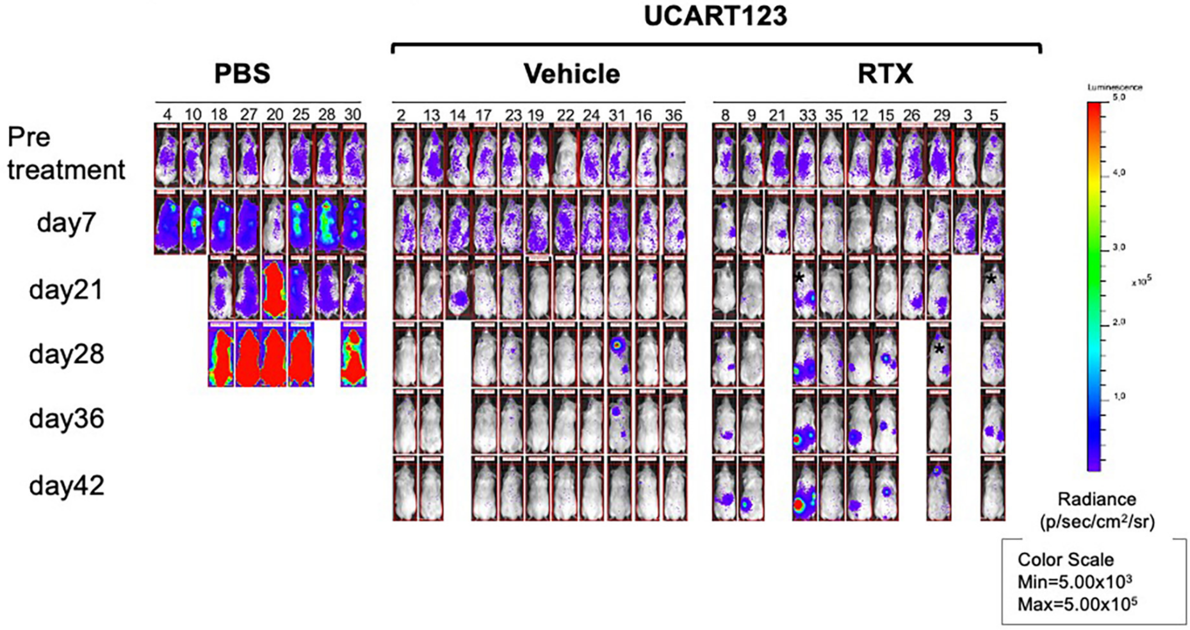

e. AML re-challenge at Day 43

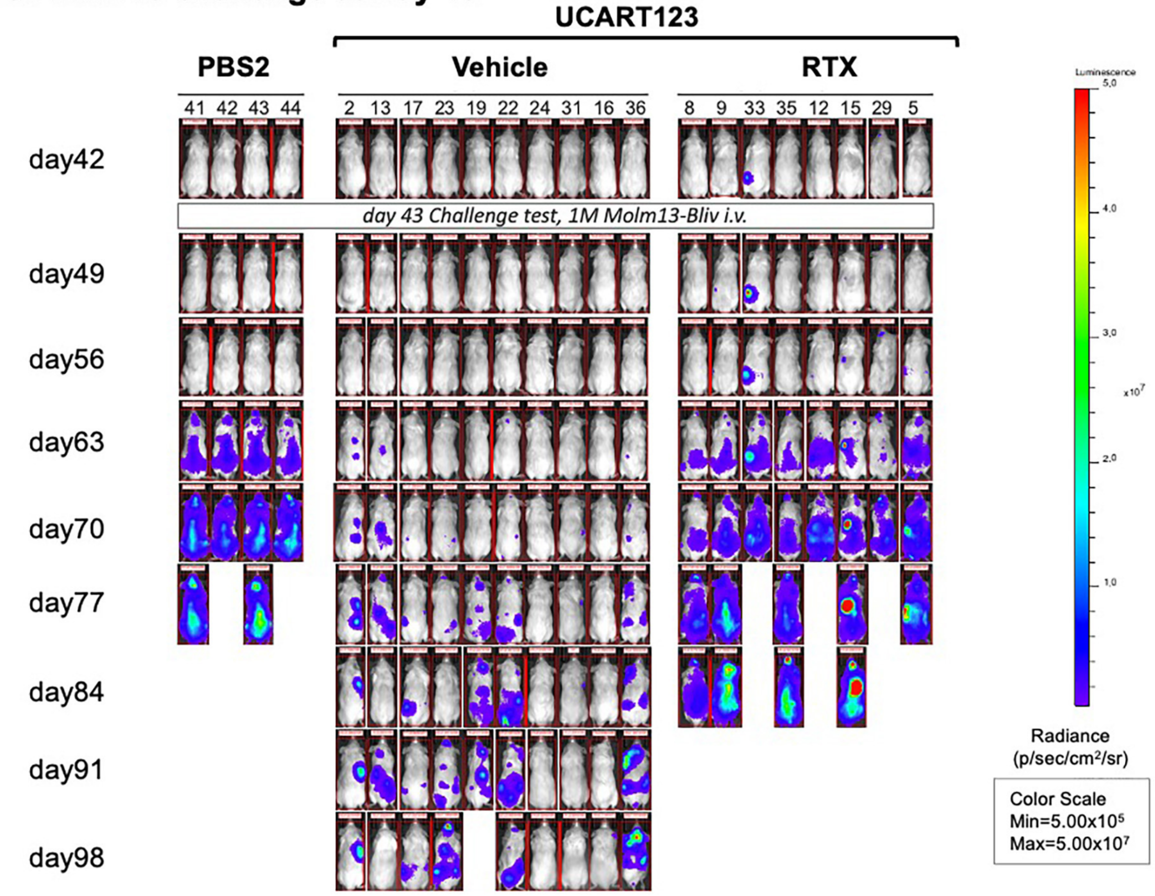

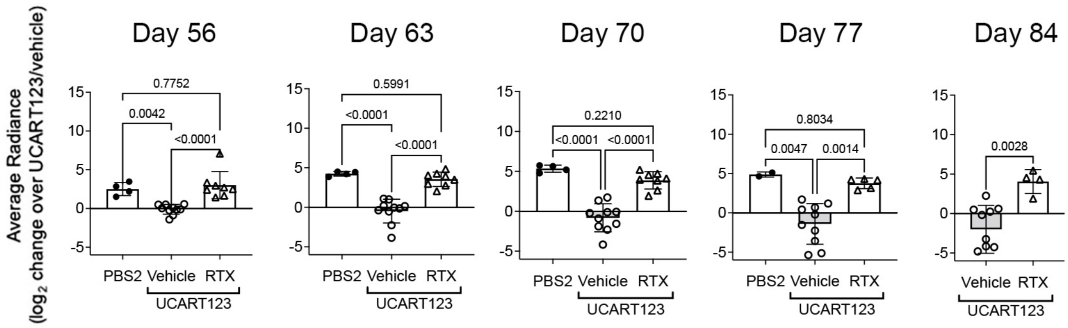

f.

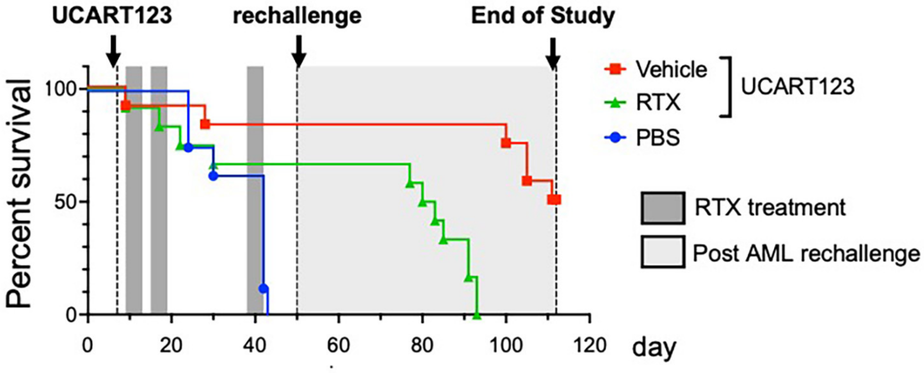

	Median survival (days)	P value vs PBS †	P value vs UCART/RTX †
UCART123	111.5	0.0010	0.0001
UCART123/RTX	81.5	0.0327	-
PBS	42	-	-
PBS2 (rechallenge)	34		

† Log rank test.

g.

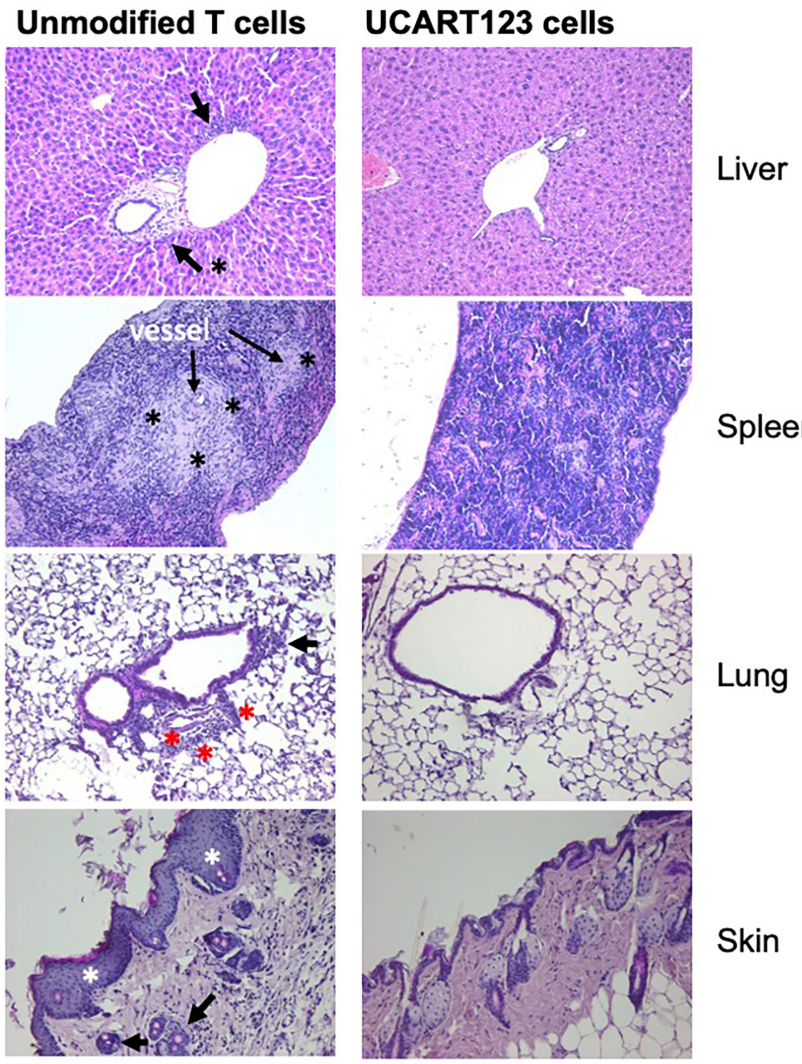

h.

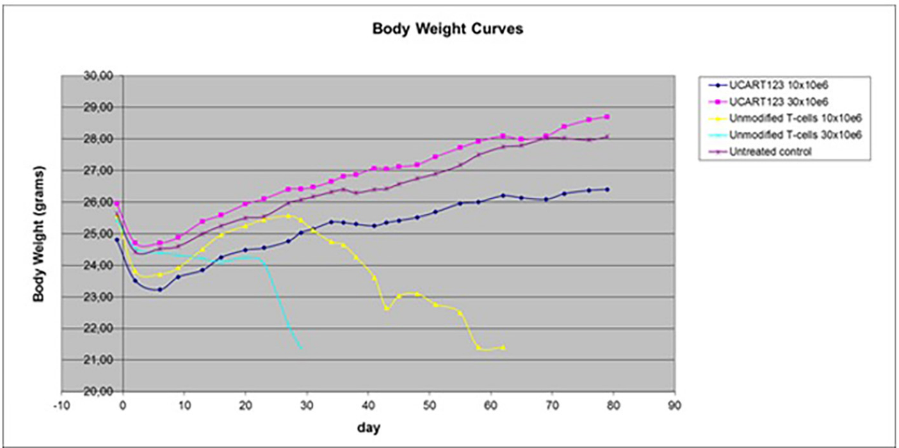

Supplementary figure 1. UCART123 features. a, anti-leukemia activity was confirmed in additional CD123+ cell lines (THP-1, KG1, Kasumi-6 and MV4;11). Cell death was evaluated at 1:1 E:T ratios after 24 h. n=2, percent death shown relative to control. **b, Effective TCRα/β KO.** Representative dot plots for the expression of the CAR and TCRα/β in the cells used for the study. **(c-f)** MOLM13-BLIV bearing mice (NSG) were treated with PBS (N=8), 10M UCART123 cells (UCART123/vehicle) (N=12) or 10M UCART123 cells followed by rituximab treatment (UCART123/RTX) n=12). Leukemia burden and UCART123 cells were monitored with in vivo bioluminescent imaging (In Vivo Imaging Systems (IVIS), PerkinElmer) and with flow cytometry (FCM). **c top,** Representative flow charts of peripheral blood samples on day 23 post UCART123 cell treatment are shown. **left,** Live single cells were gated on human CD45+ cells. **middle,** Frequency of UCART123 cells (CD5+CAR+) are shown. **right,** CD4+ and CD8+ subsets in UCART123 cells are shown. **c bottom,** Frequencies of UCART123(%) in mononuclear cells on day 2, 13, 23, and 38 were measured by FCM using PB samples from UCART123/vehicle (n=12) and UCART123/RTX (n=12). Each symbol represents one mouse and bar represents the average with the SD. **p=0.0014, ****p<0.0001, two-tailed unpaired t-Test. **d,** Leukemia burden of individual mouse measured with IVIS are shown at indicated time-points from pre-treatment to day 42 post UCART123 treatment. Mouse#11 (UCART/vehicle) and mouse #32 (UCART/RTX) died due to bleeding procedure on day 2 (not shown here). Mouse #21 (UCART/RTX) was found dead on day 10 due to technical accident after RTX injection. Mouse #26 (UCART/RTX) died due to bleeding procedure on day 23. **e,** surviving mice in UCART123/vehicle (n=11), UCART123/RTX (n=11) groups and new naïve control NSG mice (n=4, PBS2) were re-challenged on day 43 by being injected with 1 million of MOLM13-BLIV cells**. e top,** Leukemia burden of individual mouse measured with IVIS are shown at indicated time points. **e bottom,** Average radiance relative to UCART123/vehicle group are shown at indicated timepoints. Each symbol represents one mouse and bar represents the average with the SD. *P* values are calculated using ordinary one-way ANOVA Turkey's multiple comparison test (Day56, 63, 70 and 77) and two-tailed unpaired t-Test (Day 84). **p<0.01, ***p<0.001, and ****p<0.0001. **f**, survival curve of MOLM13-BLIV engrafted NSG mice treated (PBS (blue line), UCART123/vehicle (red line) and UCART123/RTX group (green line). The periods of cycles of RTX treatment are shaded in dark grey. The period from re-challenging with MOLM13-BLIV injection (day 50) to End of Study (day 112) was shaded in light grey. The median survival days of each group after MOLM13-BLIV injection are indicated in the table. *P* values UCART123 vs PBS, UCART123/RTX vs PBS and UCART123 vs UCART123/RTX are calculated by the log-rank test. **(g & h) Lack of GVHD induction;** T-cells (UCART123, or related unmodified T-cells as controls) were intravenously injected to NSG mice one day after whole body irradiation at 2Gy. Doses of $10x10^6$ or $30x10^6$ cells were tested (higher doses compared to active dose in anti-tumor studies) in 3 male + 3 female mice/group. To evaluate the impact of the irradiation, a control group where NSG mice were irradiated but not injected with T-cells was added (untreated control group). Clinical signs of GvHD were monitored (body weights were recorded twice a week and clinical observations and mortality were checked daily). **g.** Representative examples of histopathological changes in liver (periportal fibrosis (asterisk) and inflammation by mononuclear cells (arrows); spleen (perivascular fibrosis depicted by asterisks); lung (perivascular (arrows) and peribranchial (arrow) inflammation; and skin (epidermal hyperplasia (asterisks) and atrophy of adnexal sebaceous glands (arrows). Total number of animals evaluated were 3 males and 3 females per cohort. **h,** the average weight of each group was plotted. UCART123 did not affect recovery and gain of weight after irradiation, while unmodified T-cells induced weight loss due to GvHD. Source data are provided as a Source Data file.

Supplementary Figure 2

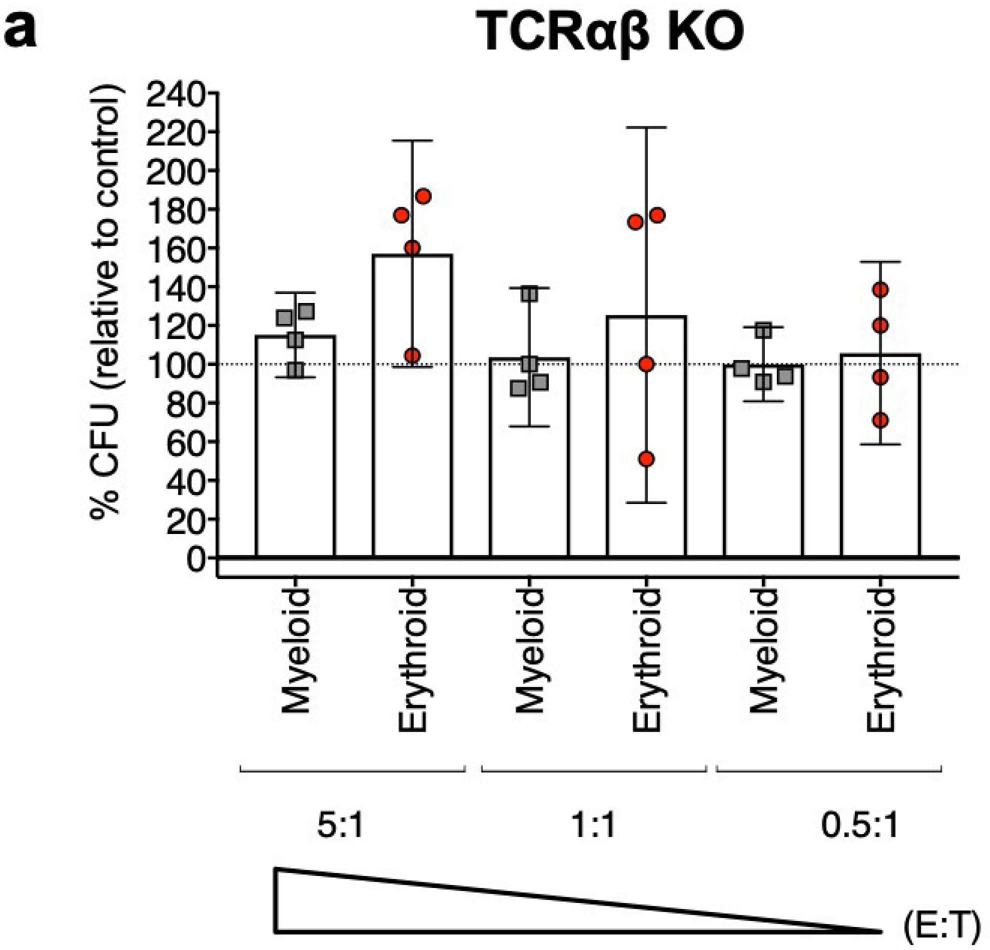

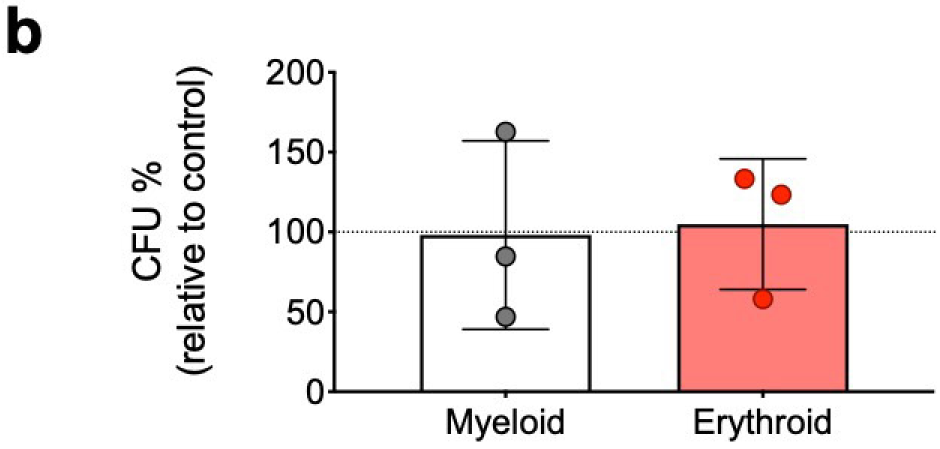

Supplementary figure 2.

a, Percent CFU relative to control of CB (n=4) samples. Cells were plated 4 hours after co-culturing with TCRαβ KO T cells at the indicated E:T ratios. Myeloid (gray) and erythroid (red) colonies are shown. **b,** Percent CFU relative to control of the secondary colony plating for 1:1 E:T ratios for CB cells treated with UCART123; n=3 Each symbol represents an individual test, and the bar represents the mean with the SD. Source data are provided as a Source Data file.

Supplementary Figure 3

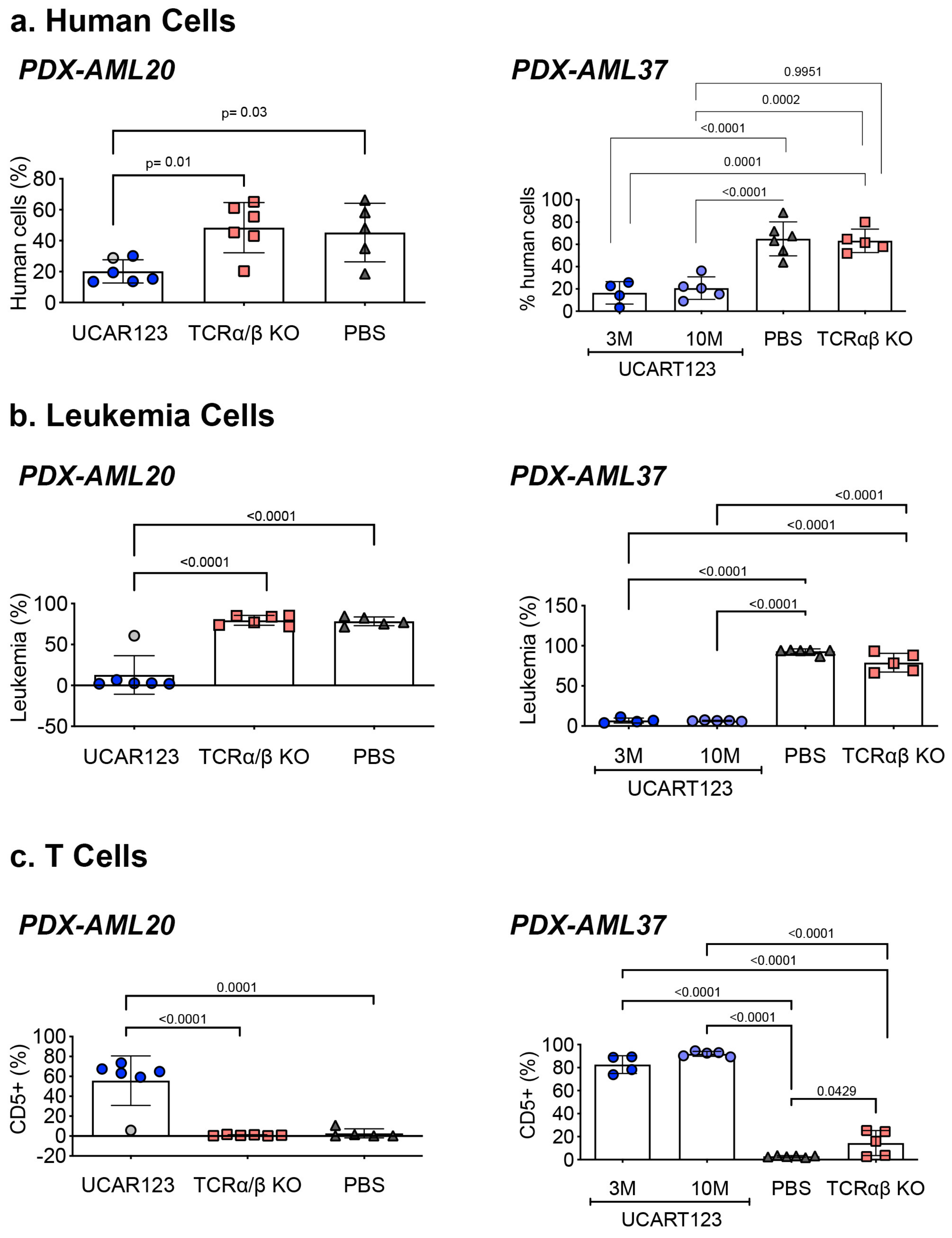

d. PB monitoring from cohorts from figure 3

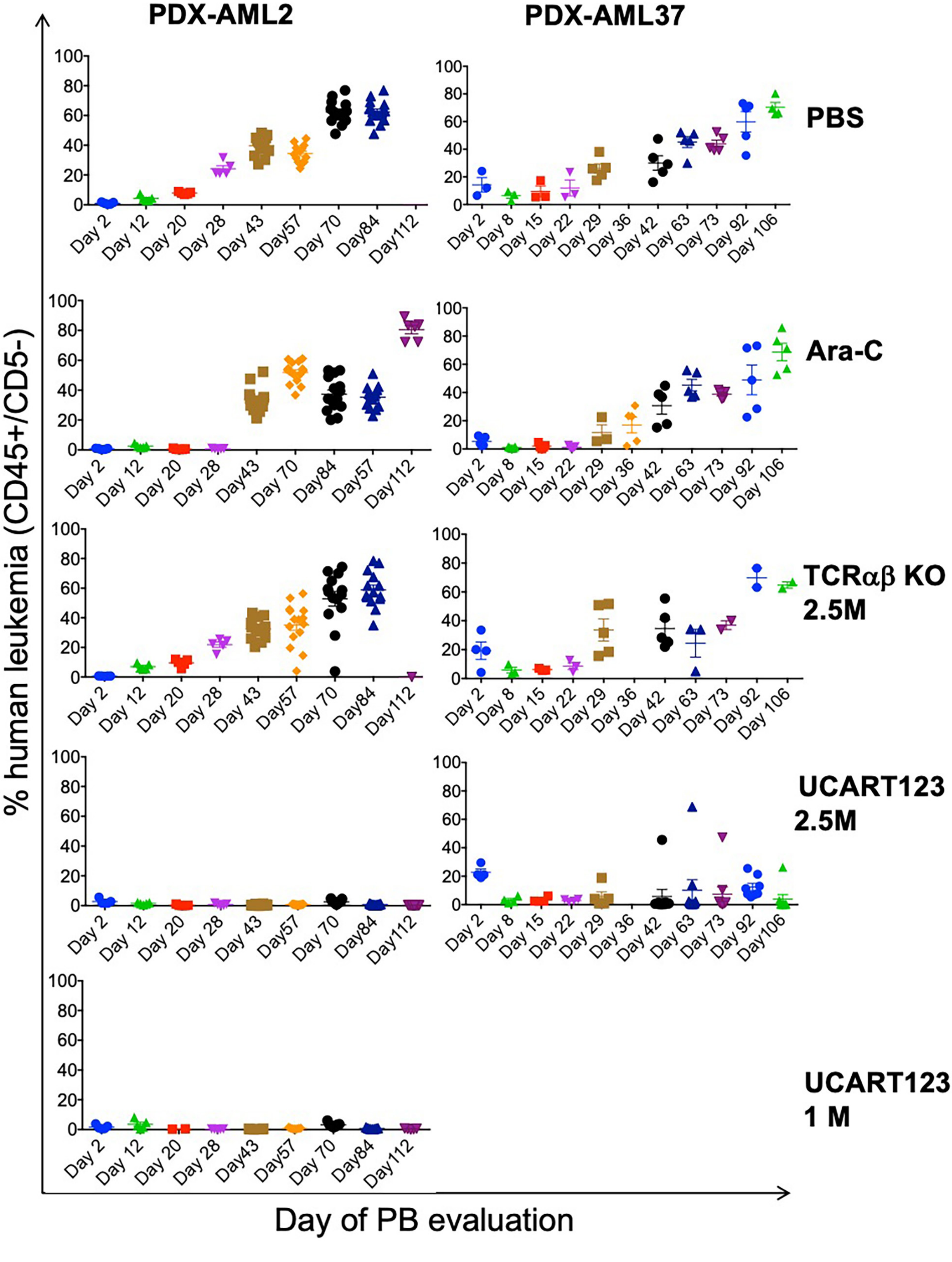

e. PDX-AML37 BM day 170

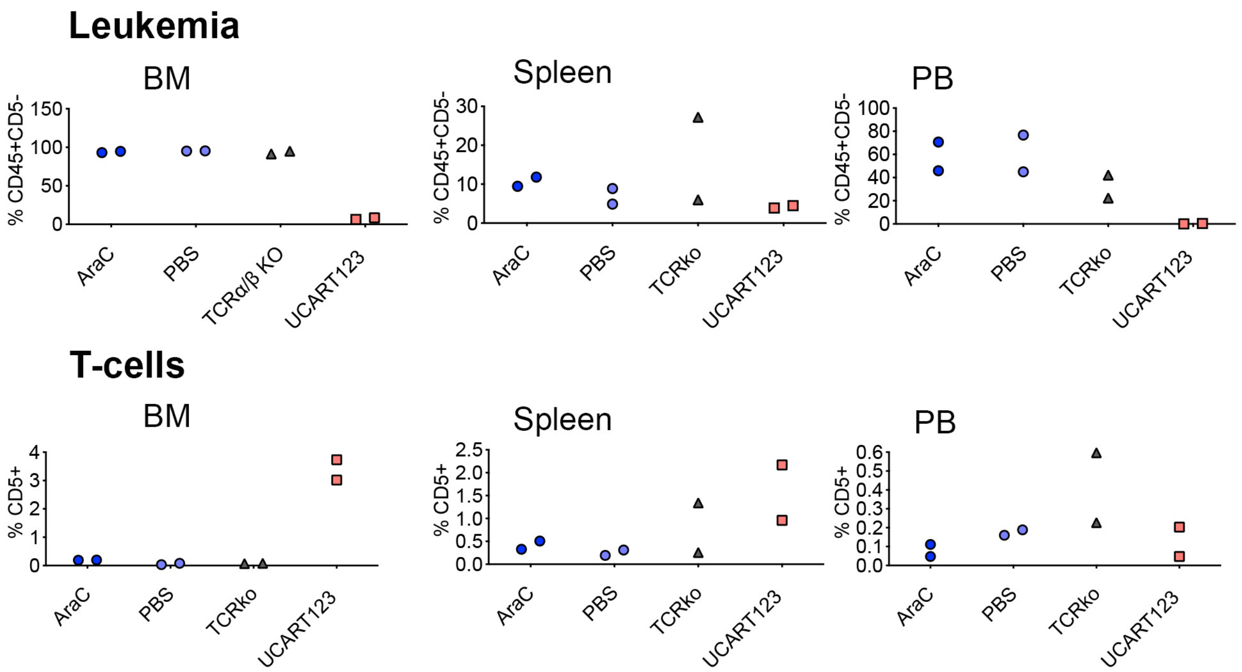

f. PDX-AML2 End of Study

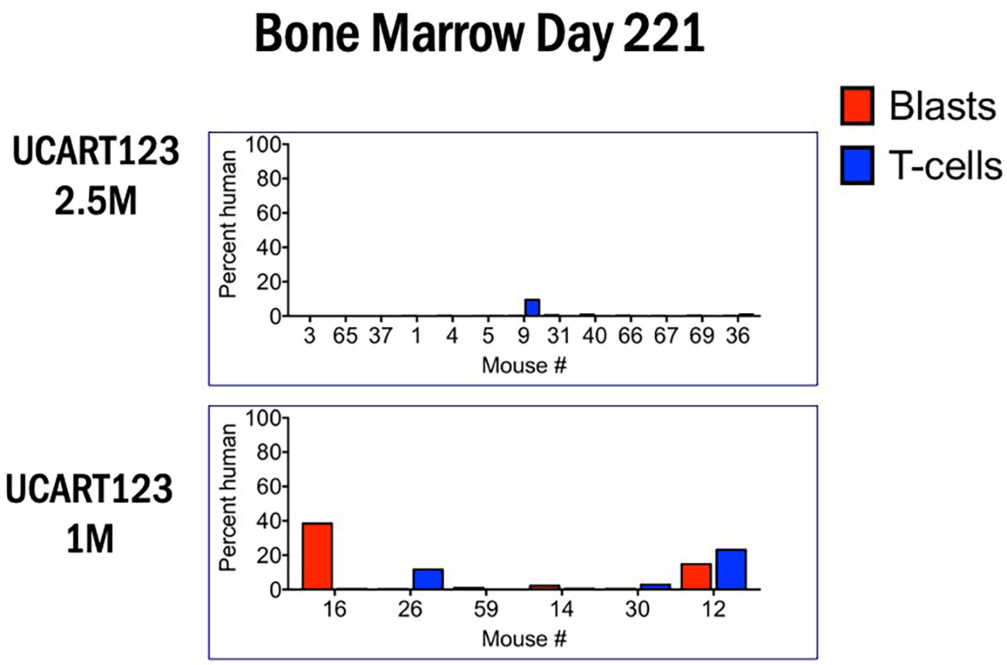

Supplementary figure 3. Assessment of leukemic burden and persistency of UCART cells in bone marrow (BM) after UCART123 treatment in PDX-AML models. (a, b, and c) PDX-AML20 mice were treated with PBS (n=5), 10×10^6 TCRαβ KO T cells (n=6), or 10×10^6 UCART123 cells (n=6) after confirmation of engraftment. and PDX-AML37 were treated with PBS (n=6), 10×10^6 TCRαβ KO T cells (n=6), 3 $\times 10^6$ UCART123 (n=4), or 3×10^6 UCART123 (n=5). Mice were sacrificed 3 weeks after treatment and cells harvested from BM were evaluated for leukemic burden and UCART123 cells by flow cytometry. UCART123 treated mice showed significant decrease of total human cells (**a**) with complete elimination of leukemia cells (**b**) and with persistent T cells (**c**), while control groups showed progressive disease. **d,** PB monitoring by flow cytometry from PDX-AML2 and PDX-AML37 cohorts in **Figure 3**. Engrafted mice with each primary AML cells were treated with PBS, Ara-C, TCRαβ KO T cells or UCART123 with indicated cell doses. PB monitoring started on day 2 and later every 1-2 weeks. UCART123 treatments significantly inhibited leukemia progression compared to controls including Ara-C and TCRαβ KO T cells cohorts in both PDX-AML2 and PDX-AML37. PDX-AML2 cohorts treated with 1 x 10^6 UCART123 (n=7), 2.5 x 10^6 UCART123 (n=15) TCRαβ KO (n=15), Ara-C (n=15) or PBS (control; n=14), and of PDX-AML37 cohorts treated with 2.5 x 10^6 UCART123 (n=7) TCRαβ KO (n=6), Ara-C (n=5) or PBS (control; n=5). **e,** For the PDX-AML37 experiment (**Figure 3c**), 2 animals from each cohort were sacrificed for evaluation of leukemic burden and persistent T cells in PB, BM and spleen on day 170. As observed in PB monitoring (**Figure 3d,** right), UCART123 mice did not have leukemia cells in BM or spleen (top row) and 3% of sustaining T cells were detected in BM of UCART123 mice (bottom row, left). **f,** The PDX-AML2 was terminated on day 221 after significant prolonged-survival. BM was evaluated for leukemic burden and persisting T cells at end of study by flow cytometry. All mice from 2.5×10^6 UCART123 cohort remained disease free (top row), while some of 1×10^6 UCART123 cohort relapsed. Each symbol represents one mouse and bar represents the mean with the SD. ****$p<0.0001$, *** $p<0.001$, **$p<0.01$, and * $p<0.05$, one-way ANOVA. Source data are provided as a Source Data file.

Supplementary Figure 4

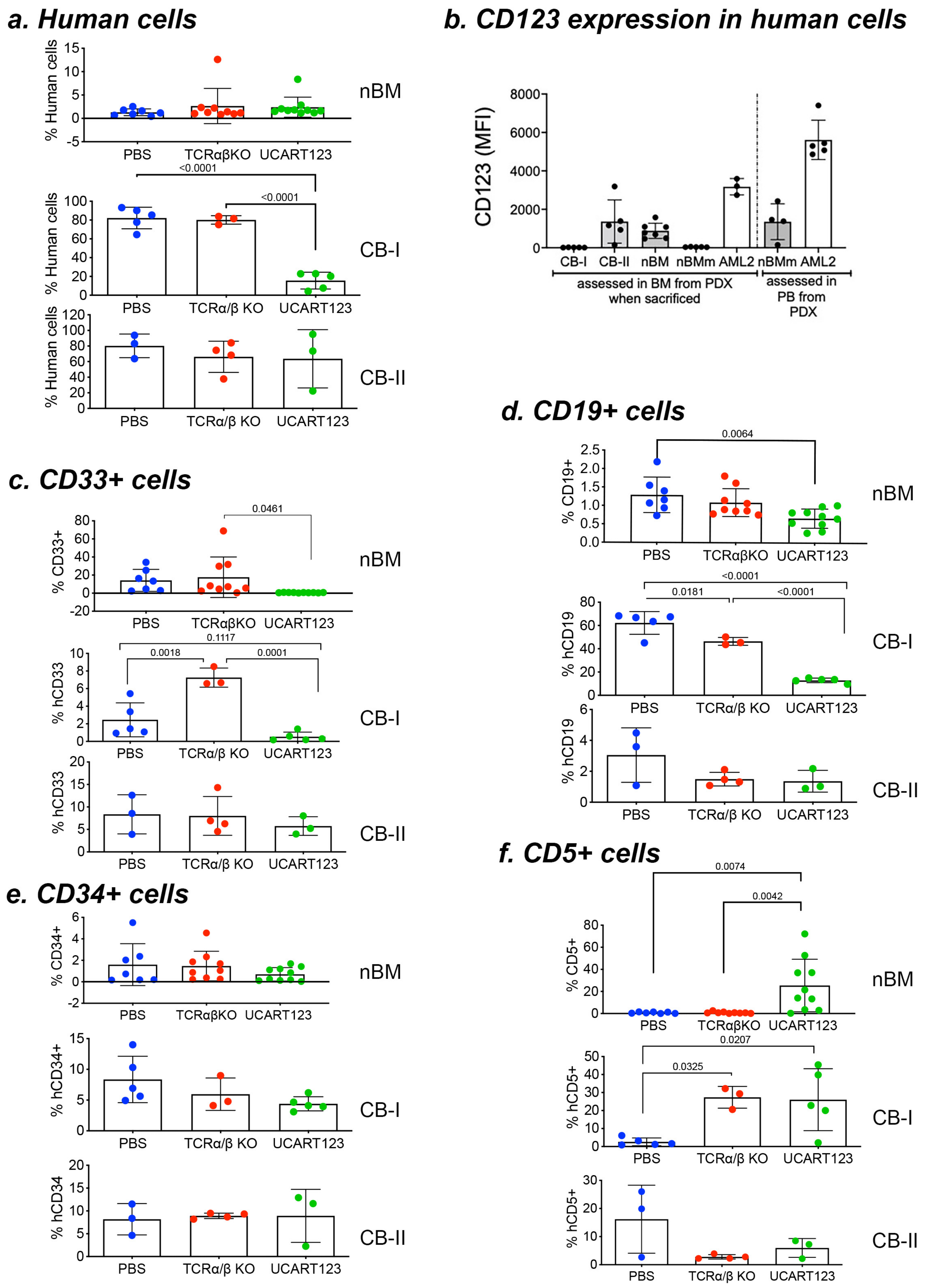

Supplementary figure 4. Evaluation of subsets after UCART123 treatment in humanized mouse with normal hematopoiesis. Humanized mice engrafted with CD34+ CB cells (CB-I, CB-II) or normal bone marrow (nBM) were treated with $10x10^6$ or $2.5x10^6$ UCART123 cells or TCRαβ KO T cells. After 1-3 months post treatment, mice were sacrificed and remaining subsets in BM were evaluated by flow cytometry. **a,** Total human cells in each of the Hu-NSG used with the indicated treatments, **b,** The mean fluorescence intensity (MFI) of CD123 measured with flow cytometry in the human leukocytes (CD45+) from the indicated Hu-NSG mice. nBMm and AML2 indicated data from nBM population and human leukemia (AML2) population in NSG co-engrafted model with nBM and AML2. Each symbol represents an animal in each of the experiments and cohorts. Bar represents the mean with the SD of all cohorts. **c- d,** changes in the proportions of myeloid (CD33, c), Lymphoid (B and T; CD19 and CD5 d,f) and hematopoietic progenitor cells (CD34+, e) were evaluated. Cohorts sizes: nBM (PBS n=10, TCRαβKO n=9, UCART123 n=10); CB-I (PBS n=5, TCRαβKO n=3, UCART123 n=5), and CB-II (PBS n=3, TCRαβKO n=4, UCART123 n=3)Each symbol represents an animal in each of the experiments and cohorts. Bar represents the mean with the SD of all cohorts. one-way ANOVA. *** *p<0.001, **p<0.01, and * p<0.05.* Source data are provided as a Source Data file.

Supplementary Table I: Characteristics of AML samples

Sample ID	Type	CD123% (blasts) 6H6	MFI	AML sample information
**AML2**	Leukapheresis	99.3	32640	Relapse; normal cytogenetics; FLT3-ITD; NPM1 mutant
**AML8**	Leukapheresis	91.1	6413	NPM1 mutant
**AML17**	Leukapheresis	99.7	27698	Relapse; normal cytogenetics; FLT3-ITD
**AML20**	Leukapheresis	47.2	3550	Diagnosis; T(11;14); FLT3-ITD
**AML33**	Leukopheresis	92.8	26943	Diagnosis;; normal cytogenetics; FLT3 point mutation
**AML34**	Leukapheresis	89.8	20340	Diagnosis; normal cytogenetics; FLT3-ITD; NPM1 mutant
**AML37**	Leukapheresis	86.1	13573	Relapse; TP53 mutant; normal cytogenetics
**AML40**	BM	86.7	12349	Diagnosis; normal cytogenetics; FLT3-ITD
**AML72**	Leukapheresis	90.4	11799	not available
**AML73**	Leukopheresis	88.2	8710	not available
**AML76**	Leukapheresis	48	14328	Monosomy 7; DNMT3A (R882H)
**AML95**	Leukapheresis	96	19041	Diagnosis; 46,XX,add(1)(p36.1), t(6;11)(q27;q23)[13]/46,XX[7]
**AML104**	BM	61.7	2620	Diagnosis;; normal cytogenetics; FLT3-ITD
**AML105**	BM	61.3	1215	Diagnosis; 3,-5,del(5)(q13q33),+8,+2～4mar[cp20]; TP53 (c.365_366delTG;p.V122Dfs*26)

Supplementary Table 2. Characteristics of T cells

Process	Cell type	%CAR+	%CD8+	%CD4+	%Cytotoxicity	%TCRab+
**Pr67**	UCART123 cells (R&D, large scale)	90.4	44.8	54.2	60.5	0.2
**Pr62**	UCART123 cells (R&D, large scale)	90.5	58.7	36	60	0.4
**Pr67 (UTD/KO)**	TCRαβ-deficient T-cells (TCRαβ KO; R&D, Large Scale)	N/A	32.7	37.3	0	0.92
**Pr66 (UTD/KO)**	TCRαβ-deficient T-cells (TCRαβ KO; R&D, Large Scale)	N/A	57.7	38.4	0	0
**UCART123 A4749**	UCART123 Cells (R&D, small scale)	95.9	77.9	13.9	54	8.2
**UTD/KO A4749**	UCART123 Cells (R&D, small scale)	N/A	32.9	39	0	9.2
**UCART123 A4739**	UCART123 Cells (R&D, small scale)	88.7	57.8	34.4	13.7	8.5
**UTD/KO A4739**	UCART123 Cells (R&D, small scale)	N/A	37.7	45.5	0	8

% CAR+ represents the percentage of CD45+ / CD4 or CD8+ / CAR+ / TCRαβ- cells over the CD45+ cells at Day19 (end of production process, before freezing). Note that total of CD8+ and CD4+ cells is not equal to 100% because of the presence of several double negative CD8- CD4- cells.

% CD8+ represents the percentage of CD45+ / CD8+ over the CD45+ cells at Day19.

% CD4+ represents the percentage of CD45+ / CD4+ over the CD45+ cells at Day19.

% cytotoxicity: Activity using in vitro cytotoxicity assay after thawing determined by CLS against MOLM-13 cells.

Supplementary Table 3.

	mouse ID	total MNCs	hCD45+	CAR+ UCART123
UCART123	2	20,230	76	62
	13	15,542	44	35
	16	16,370	54	45
	17	11,350	64	44
	19	10,793	41	26
	22	17,886	150	140
	23	24,840	54	46
	24	39,360	96	82
	31	28,400	76	66
	36	16,405	48	39
UCART123/RTX	5	12,844	14	4
	8	96,591	18	2
	9	37,733	29	5
	12	27,880	24	3
	15	24,812	56	11
	26	26,731	31	8
	29	18,465	37	6
	33	5,728	9	3
	35	18,952	34	9
Saline	18	35,290	69	0
	20	11,529	43	0
	25	20,302	57	0
	27	31,795	117	0
	28	43,993	154	0
	30	31,853	48	0

Supplemental Table 3. Event numbers are indicated that were acquired with flow cytometry performed with isolated cells from 50uL peripheral blood on day 23 post UCART123 treatment in Molm13-Bliv engrafted NSG mice.
*UCART; UCART123, RTX; Rituximab.

Supplementary Table 4. Antibodies used

Epitope	Fluorchrome	Species	Dilution	Clone	Catalog number	Company
**CD45**	APC-H7	human	1:100	2D1	560178	BD Pharmingen
**CD45**	FITC	human	1:20-1:50	HI30	555482	BD Pharmingen
**CD45**	PE	human	1:200	HI30	555483	BD Pharmingen
**CD38**	PECy7	human	1:100	HIT2	303516	eBioscience
**CD34**	APC	human	1:100	8G12	340441	BD Bioscience
**CD34**	APC	human	1:100	4H11	17-0349-42	eBioscience
**CD34**	PeCY7	human	1:100	8G12	348791	BD Bioscience
**CD34**	PECY5	human	1:100	581	555823	BD Pharmingen
**CD34**	PE Dazzle 594	human	1:100	Clone 581	343534	BioLegend
**CD33**	BB515	human	1:100	WM53	564588	BD Horizon
**CD5**	APC	human	1:100	UCHT2	561003	BD Bioesciece
**CD8**	BV785	Human	1:200	SK1	344740	BioLegend
**CD8**	BV510	human	1:100	RPA-TB		BD Bioesciece
**CD8**	PE	Human	1:200	BW 135/80	130-091-084	MACS
**TCR a/b**	Vioblue (V450)	Human	1:50	BW242/412	130-098-783	Miltenyi Biotec
**CD123**	PE	human	1:40	6H6	12-1239-42	eBioscience
**CD123**	BV650	human	1:40	6H6	306019	BioLegend
**CD123**	PerCPCy5.5	human	1:20	7G3	558714	BD Pharmingen
**CD123**	PerCpCy5.5	human	1:40	6H6	306016	BioLegend
**HLA A2**	PE	Human	1:2000	BB7.2	343306	BioLegend
**HLA A2**	AF700	Human	1:50	BB7.2	343318	BioLegend
**CD34**	PE	Human	1:50	QBEnd10	FAB7227P	R&D systems
**CD34**	APCH7	Human	1:50	QBEnd10	FAB7227A	R&D systems
**CD34**	AF488	Human	1:50	QBEnd10	FAB7227G	R&D systems
**CD3**	APCH7	Human	1:100	SK7	51-9007098	BD Pharmingen
**CD3**	BV785	human	1:100-1:200	OKT3	317330	BioLegend
**CD4**	PerCPCy5.5	Human	1:50	SK3	51-9007099	BD Pharmingen
**CD197(CCR7)**	AF647	Human	1:100	150503	51-9007097	BD Pharmingen
**CD19**	PE	human	1:50	HIB19		eBioscience
**CD45RA**	FITC	Human	1:50	HI100	51-9007100	BD Pharmingen
**CD197(CCR7)**	AF647	Human	1:50	150503	51-9007097	BD Pharmingen
**CD25**	AF700	human	1:25	M-A251	561398	BD Pharmingen
**CD56**	AF700	human	1:50	B159	557919	BD Bio
**CD19**	PE	human	1:200	HIB19	12-0199-42	eBioscience
**CD19**	PerCpCy5.5	human	1:100	HIB19	302230	BioLegend
**CD71**	AF700	human	1:50	M-A712	563769	BD bioScience
**CD133/2**	APC	human	1:50	293C3	130-090-854	MACS
**CD107a**	APC	Human	1:500	H4A3		BD Fastimmune
**CD14**	PE	human	1:100	M5E2	555398	BD Pharmingen
**CD56**	PE	human	1:50	B159	555516	BD Pharmingen
**CD56**	AF700	human	1:50	B159	557919	BD Biosciences
**anti mouse IgG**	PE	mouse	1:5000	n/a	115-115-164	Jackson ImmunoResearch
**anti mouse IgG**	APC	mouse	1:500-1:1000	n/a	115-135-164	Jackson ImmunoResearch
**human BD Fc Block**	n/a	human	1:40-1:200	n/a	564220	BD Pharmingen
**Annexin-V**	PE	n/a	1:100	n/a	556421	BD Pharmingen
**CD45**	PECy5	mouse	1:5000	30-F11	103110	BioLegend

Supplementary Table 5. Primers and Probes

Target	Primers and probe sequece	Reference
CAR-F	5-GAGCTGAGGGTCAAGTTTAG -3	
CAR-R	5-TTATCCAGCACGTCGTATTC -3	
CAR_probe	5-6FAM-CCG TCC CAG-ZEN-ATT CAG CTC GTT ATA C-3IABkFQ-3	
ABL1-ENF1003	5-TGGAGATAACACTCTAAGCATAACTAAAGGT-3	[1]
ABL1-ENR1063	5-GATGTAGTTGCTTGGGACCCA-3	
ABL1-1043V-MGB	5-VIC-CATTTTTGGTTTGGGCTTC-MGB-3	
NPM1-common-F	5-GAAGAATTGCTTCCGGATGACT-3	[1]
NPM1-multiplex-R *	5-CTTCCTCCACTGCNNNNCAGA-3	
NPM1_probe	5-FAM-ACCAAGAGGCTATTCAA-MGB-3	

References:

1. Mencia-Trinchant, N. *et al.* Minimal Residual Disease Monitoring of Acute Myeloid Leukemia by Massively Multiplex Digital PCR in Patients with NPM1 Mutations. *J Mol Diagn* **19**, 537-548, doi:10.1016/j.jmoldx.2017.03.005 (2017).
